# Fungal Zinc Homeostasis and Its Potential as an Antifungal Target: A Focus on the Human Pathogen *Aspergillus fumigatus*

**DOI:** 10.3390/microorganisms10122469

**Published:** 2022-12-14

**Authors:** Pengfei Zhai, Yanfei Chai, Ling Lu

**Affiliations:** 1 Jiangsu Key Laboratory for Microbes and Functional Genomics, College of Life Sciences, Nanjing Normal University, Nanjing 210023, China; 2 Center for Hygienic Analysis and Detection, Nanjing Medical University, Nanjing 211166, China

**Keywords:** *Aspergillus fumigatus*, zinc transporter, zinc homeostasis, therapeutic target, virulence

## Abstract

*Aspergillus fumigatus* is an opportunistic airborne fungus that causes severe invasive aspergillosis in immunocompromised patients. Zinc is an essential micronutrient for the growth of *A. fumigatus* and even for all microorganisms. An increasing number of studies have reported that fungal zinc acquisition ability plays a key role in fungal survival in hosts with an extremely zinc-limited microenvironment. The ability to fight scarcity and excess of zinc are tightly related to fungal virulence and may be used as new potential targets. Because the regulation of zinc homeostasis is important, a thorough understanding of the functional genes involved in the regulatory network for zinc homeostasis is required for fungal pathogens. The current mini-review summarized potential zinc homeostasis regulators in *A. fumigatus* and classified these regulators according to localization and function, which were identified or predicted based on *A. fumigatus* or deduced from homologs in model yeasts. Future perspectives for zinc homeostasis regulators as potential antifungal targets to treat invasive aspergillosis are also discussed.

## 1. Introduction

*Aspergillus fumigatus* is an opportunistic and saprophytic filamentous fungal pathogen that proliferates in the lungs of immunocompromised patients and causes invasive pulmonary aspergillosis, which is an infectious disease with a fatality rate of up to 95% [1,2,3,4,5]. There are three main clinical antifungal drugs, polyenes, azoles and echinocandins, and azoles are the first-line antifungal drugs in clinical treatment [6]. However, a large number of azole-resistant *A. fumigatus* isolates have been found in many countries in recent years [7,8,9]. The limitations of these antifungal drugs, such as side effects and toxicity, make the development of new strategies targeting virulence factors imperative to improve efficacy against pathogenic targets.

The virulence of *A. fumigatus* relies on its pathogenicity and host immune capacity, but its pathogenicity is multifactorial and sophisticated [10]. The host actively reduces the nutritional availability of the pathogen, and the mechanism of nutrient uptake is crucial in fungal infections. The ability to acquire zinc as a nutrient is an important factor in fungal pathobiology and virulence [11,12,13]. Zinc is the second most abundant trace metal in human nutrition, after iron, and it is a key component in maintaining the stability of many proteins. For example, Zn is a cofactor for six major functional enzyme classes (cleavage enzymes, isomerases, ligases, oxidoreductases, transferases and hydrolases) that assists in catalytic and co-catalytic activities, and it is necessary for the activity of many transcription factors [14,15,16]. Therefore, zinc plays a vital role in cellular processes and diseases, such as growth and development, transcription, translation, gliotoxin biosynthesis and mycetoma [17,18,19,20,21,22,23]. Many studies have shown that hosts have evolved an antimicrobial strategy based on microbial zinc limitation within neutrophils [24,25,26]. The concentration of free Zn^2+^ is closely related to the pH of the environment, and it readily forms complexes with other compounds as the pH increases [27]. Therefore, the concentration of free Zn^2+^ in the host body at physiological pH (7.3–7.4) is very low because most of the zinc in intracellular and extracellular fluids is firmly bound to zinc-binding proteins [28,29]. The free zinc concentration in humans is approximately 150-fold lower than the minimum concentration required for fungal growth [30]. When the host encounters fungal infection, the Zn/Mn chelating protein calprotectin is released by neutrophils to reduce Zn^2+^ and Mn^2+^ utilization and inhibit microbial growth [31,32,33]. Zinc availability is further reduced by the competitive use of zinc by pulmonary bacteria, and immunodeficient patients generally experience mixed microbial infections [34]. Therefore, genes involved in the regulation of zinc homeostasis become particularly important for fungal virulence. Because of these crucial roles for Zn in fungal pathogenicity, it is necessary to summarize recent findings regarding zinc homeostasis of *A. fumigatus* to promote the search for new therapeutic targets and the development of antifungal drugs to fight fungal pathogens.

## 2. Members Involved in Zinc Homeostasis in *A. fumigatus*

### 2.1. Identified or Predicted Transporters Involved in Zinc Transport at the Plasma Membrane

The zinc uptake system in the fungal plasma membrane facilitates the passage of zinc through the plasma membrane into the cytoplasm to participate in a variety of cellular physiological processes. Our understanding of zinc homeostasis within the fungus *A. fumigatus* primarily comes from results in the model yeast *Saccharomyces cerevisiae* [35,36,37]. The protein family Zrt-/irt-like protein (ZIP) tightly controls Zn acquisition in *S. cerevisiae* [38]. The ZIP family is derived from the Zrt1 protein of *S. cerevisiae* and the Irt1 protein of *Arabidopsis thaliana*, and it belongs to the solute carrier protein SLC39A family [39,40]. Zrt1 and Zrt2 have been identified as members of the ZIP family with plasma membrane localization in *S. cerevisiae* [41]. These protein family members are responsible for the transport of metal ions, such as zinc, from the extracellular environment or organelle cavities to the cytoplasm. Among the *S. cerevisiae* ZIP transporters, Zrt1 (zinc-regulated transporter) was first identified as a high-affinity transporter protein for zinc uptake under zinc-deficient conditions [42,43,44]. Zrt2 was previously reported to be a low-affinity transporter that only transported zinc under mild zinc-limited conditions [37,45]. ZIP family members are highly conserved across fungal species, and there are eight putative ZIP family proteins in *A. fumigatus* (Table 1): ZrfA (AFUB_079250), ZrfB (AFUB_020930), ZrfC (AFUB_066680), ZrfD (AFUB_097050), ZrfE (AFUB_083560), ZrfF (AFUB_024650), ZrfG (AFUB_018540) and ZrfH (AFUB_027750) [33,41]. Proteins with presumed plasma membrane localization are ZrfA, ZrfB, ZrfC, ZrfD and ZrfE. ZIP transporters have corresponding homologous proteins in the yeast *S. cerevisiae*, except the ZrfC, ZrfD and ZrfE proteins.

**Figure 1:**
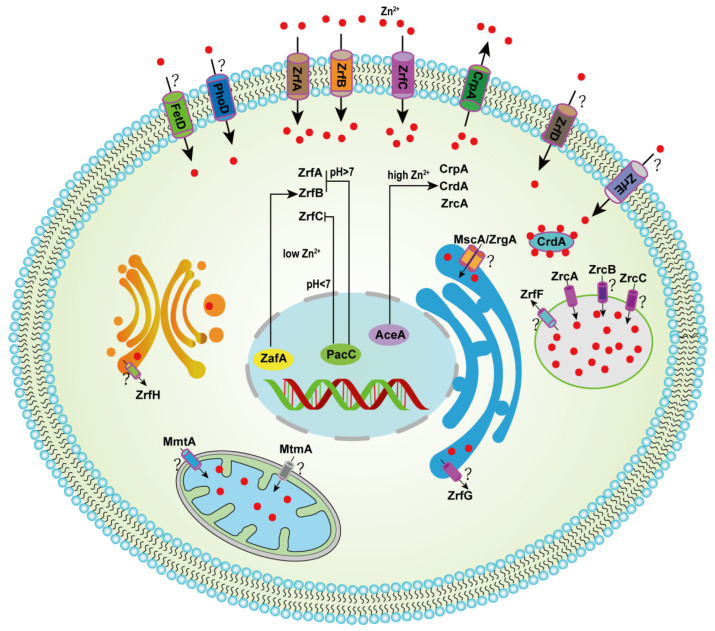
Hypothetical model for the homeostatic mechanism of zinc in *A. fumigatus*. Under acidic pH conditions, zinc enters the cytoplasm from the environment mainly through uptake by the transporters ZrfA and ZrfB, and under neutral to alkaline conditions, zinc enters the cytoplasm mainly through uptake by the transporter ZrfC. The transcription factor ZafA induced the expression of ZrfA, ZrfB and ZrfC in low-zinc conditions, and PacC suppressed ZrfA/B under alkaline conditions and ZrfC under acidic pH. Transcription factor AceA, under high-Zn conditions, induces the expression of CrpA, CrdA and ZrcA, which are transported to the extracellular space and vacuoles, respectively. The symbol “?” indicates unexplored functions of the gene involved in zinc homeostasis.

The Zn transport function of three of these eight transporters in *A. fumigatus,* ZrfA, ZrfB and ZrfC, have been deeply studied [30,46,47]. ZrfA and ZrfB are homologous proteins of *S. cerevisiae* Zrt1 and Zrt2, respectively, but ZrfC, which is also located on the plasma membrane, lacks homologs in *S. cerevisiae* [30,46]. Notably, *A. fumigatus* proliferates and grows in a wider pH range than *S. cerevisiae*, especially under alkaline conditions. Therefore, *A. fumigatus* has developed various pathways to acquire zinc, especially under acidic or alkaline zinc-limited environmental conditions. ZrfA and ZrfB are primarily responsible for zinc uptake under acidic Zn-limiting conditions in *A. fumigatus*, and ZrfC transports zinc into the cytoplasm under neutral to alkaline Zn-limiting conditions, which allows it to survive under different Zn-limiting conditions (Figure 1) [30,46]. The expression of *zrfA* and *zrfB* is zinc-dependent, and these genes are differentially significantly downregulated with increasing concentrations of zinc ions [46]. Therefore, the deletion of *zrfB* resulted in greater growth defects than the deletion of *zrfA*, and the double deletion of these two genes resulted in more severe growth defects than the deletion of *zrfB* under acidic zinc-limited conditions [46]. The transcription of these two genes is downregulated to some extent in neutral or slightly alkaline zinc-limited conditions. The transcript levels of these two genes remained significantly reduced even with additional zinc ion supplementation. Notably, the growth of the *zrfA* and *zrfB* double mutant strains was seriously affected in an acid zinc-deficient environment, but the phenotypic characterization was similar to the wild-type strain in an alkaline zinc-deficient environment [46]. These results indicate that ZrfA and ZrfB primarily play a role in acid zinc-limiting conditions. To effectively acquire zinc nutrition from the host and maintain its growth, pathogenic *A. fumigatus* uses two acidic zinc transporters, ZrfA and ZrfB, and the ZrfC alkaline zinc transporter [30,47,48]. ZrfC has a long N-terminus that contains four putative zinc-binding motifs that are not present in ZrfA or ZrfB, and the ability to obtain zinc under alkaline conditions seems to depend on it [30,48]. The role of ZrfD and ZrfE in the transport of zinc in *A. fumigatus* has not been clearly investigated, and further studies are needed for a more comprehensive understanding of zinc homeostasis.

Fet4 is also an important low-affinity transporter protein for iron and copper, and it may be involved in zinc transport in *S. cerevisiae* [49,50]. The high-affinity phosphate transporter protein Pho84 plays a role in maintaining phosphate homeostasis, but it is also involved in zinc uptake in *S. cerevisiae* [51]. However, whether the homologous proteins FetD (homolog of Fet4) and PhoD (homolog of Pho84) in *A. fumigatus* are involved in the transport of Zn is not known, and further studies are needed.

As mentioned above, fungi have developed complex countermeasures to deal with zinc deficiency swiftly. However, excess zinc also needs corresponding sophisticated mechanisms for quick detoxification. Recent studies found that the P-type ATPase CrpA was important for copper resistance and zinc detoxification in *A. fumigatus* [52,53,54]. CrpA has been identified as an exporter of zinc, in contrast to the ZIP family of importers, and *A. fumigatus* uses CrpA primarily to efflux excess zinc (Figure 1). Cell membrane-localized CrpA is involved in the intracellular-to-extracellular transport of zinc, and *crpA* deletion causes a growth defect in *A. fumigatus* that is sensitive to high zinc conditions [52].

### 2.2. Potential Zinc Transport-Involved and Organelle-Localized Transporters

Zinc transporters on organelles also play important roles in zinc homeostasis. *S. cerevisiae* has three ZIP family proteins located on organelles, Zrt3, Yke4 and Atx2 [41]. Zrt3 is a vacuole membrane transporter protein that mobilizes zinc in vacuoles to stabilize intracellular zinc ion equilibrium [37]. Yke4 is located in the endoplasmic reticulum (ER) membrane and plays a significant role in maintaining the balance of zinc ions between the cytoplasm and secretory pathways [55]. The ZIP family protein Atx2 is localized to the Golgi apparatus membrane, and it is a manganese transporter that regulates the intracellular manganese balance and inhibits oxidative stress in the absence of copper/zinc superoxide dismutase (SOD1) [56,57]. However, whether Atx2 plays a role in zinc ion homeostasis has not been reported. Although Zrt3, Yke4 and Atx2 have been demonstrated in yeast, the function of these three proteins as transporters in the homologs of *A. fumigatus* (ZrfF, ZrfG and ZrfH, respectively) has not been demonstrated. Therefore, their roles in zinc transport in *A. fumigatus* must be further investigated.

Another transporter family that plays an important role in zinc homeostasis is the cation diffusion facilitator (CDF) family, which contributes to the transport of excess metals from the cytoplasm to intracellular organelles [58,59]. The representative proteins of the CDF family in yeast are Zrc1, Cot1, Zrg17, Msc2 and Mmt1/2. Within fungal cells, vacuoles are the main organelles for the storage and detoxification of superfluous ions, such as zinc. Data on the detoxification of zinc in vacuoles in *S. cerevisiae* indicate that this process depends on Zrc1 and Cot1 zinc importers on the vacuolar membrane [60,61,62,63]. Compared to *S. cerevisiae*, *A. fumigatus* has three putative vacuolar CDF transporters, ZrcA (AFUB_092140), ZrcB (AFUB_098870) and ZrcC (AFUB_030200). Of these three CDF transporters, ZrcC has the highest homology to the yeast Zrc1/Cot1 protein, while ZrcA responded to high-Zn stimulation [52,64]. A ZrcA-deleted strain is hypersensitive to excess Zn, which suggests that ZrcA plays an important role in the detoxification of Zn in *A. fumigatus* [52]. However, whether ZrcB and ZrcC have roles as exporters in transporting zinc has not been verified.

Two other CDF family members, Zrg17 and Msc2, localize to the ER membrane and compose a heteromeric complex that imports zinc into the endoplasmic reticulum for proper protein processing [65,66]. The CDF family members Mmt1 and Mmt2 are mitochondrial iron ion exporters, but the function of Mmt1/Mmt2 in zinc export has not been reported [67,68]. Although proteins Zrg17, Msc2 and Mmt1/2 have only been demonstrated in yeast, homologs of all three proteins are encoded in the genome of *A. fumigatus,* ZrgA (AFUB_011560), MscA (AFUB_000580) and MmtA (AFUB_057390), respectively, but their functions as CDF family proteins have not been investigated. In contrast to *S. cerevisiae*, there are two other unique CDF family proteins, MtpA (AFUB_013980) and MtpB (AFUB_097080), in *A. fumigatus*, whose functions have also not been investigated. Therefore, further studies on the function of CDF protein family members are required to supplement and perfect the zinc detoxification mechanism of *A. fumigatus.*

### 2.3. Zinc-Responsive Transcription Factors

Zinc homeostasis in fungi is primarily regulated at the transcriptional level via zinc-responsive transcription factors. Zinc starvation in *S. cerevisiae* is sensed by the zinc-responsive transcription factor Zap1, which activates Zrt1/2 of the plasma membrane and Zrt3 of the vacuolar membrane in response to zinc starvation under zinc-limited conditions, regardless of the ambient pH [69,70]. Similar to *S. cerevisiae*, *A. fumigatus* zinc-responsive transcription factor ZafA is a potential homolog of *S. cerevisiae* Zap1 that governs the expression of zinc uptake genes [71,72]. Unlike *S. cerevisiae*, the expression of the zinc transporters in *A. fumigatus*, ZrfA, ZrfB and ZrfC, is influenced by the environmental pH and the zinc concentration. Therefore, *A. fumigatus* has evolved delicate mechanisms to effectively obtain the necessary zinc from acidic or alkaline environments. Under acidic zinc-limiting conditions, the *A. fumigatus* transcription activator ZafA combine with zinc response (ZR) motifs in the *zrfA* and *zrfB* promoters to induce their expressions [72]. Under acidic zinc-limiting conditions, transcription factor ZafA also binds to the *zrfC* promoter, but ZrfC expression is inhibited by another transcription factor, PacC [47]. PacC is a pH-responsive transcription factor that activates gene expression under alkaline conditions and inhibits gene expression under acidic conditions [73,74]. The expression of *zrfA* was partially inhibited under alkaline zinc-limiting conditions, and the expression of *zrfB* was strongly inhibited by the transcriptional regulator PacC [74]. Under alkaline zinc-limiting conditions, the inhibition of PacC was overcome by the transcriptional activation of ZafA, which strongly increased the expression of *zrfC* [47]. In summary, *A. fumigatus* has evolved a more refined regulatory mechanism to regulate zinc homeostasis compared to yeast.

Although zinc is required for fungal growth, excess zinc is potentially toxic to fungal cells. Therefore, fungi also have high levels of zinc-responsive transcription factors that respond to excess zinc. The copper-responsive transcription factor AceA in *A. fumigatus* is involved in copper detoxification and plays an important role in zinc detoxification [52]. However, whether the homologous proteins of AceA in other fungi are involved in zinc detoxification has not been demonstrated. Phylogenetic analysis revealed that most AceA homologs in fungi had conserved N-terminal features, including a zinc module and a copper-regulatory domain, which suggest that this copper-responsive transcription factor plays a common zinc detoxification role in fungi [52]. When *A. fumigatus* is exposed to high Zn conditions, AceA significantly induces the expression of the plasma membrane efflux pump CrpA and the vacuolar transporter ZrcA. Although AceA also induces the expression of the metallothionein CrdA in response to high Zn, *A. fumigatus* primarily uses CrpA and ZrcA, rather than CrdA, as zinc-resistance mechanisms [52]. However, the current knowledge of AceA for targeted gene binding motifs is limited, and the mechanism of AceA involvement in the regulation of zinc detoxification has not been explored in *A. fumigatus*. Therefore, the identification of the binding motifs of AceA will be the priority of future studies in *A. fumigatus*.

### 2.4. Zinc Trafficking-Involved and other Zinc Homeostasis-Related Proteins

Besides zinc transporters and transcription factors involved in zinc homeostasis, there are other types of proteins involved in metal homeostases, such as zincophores, metallothioneins and metal chaperones. The zinc homeostatic system that involves the allergen Aspf2 is important for the survival of *A. fumigatus* in alkaline and extreme zinc-limited environments [47,75]. A recent study showed that the C-terminus of Aspf2 had specific Zn-binding sites that acted as zincophores to deliver Zn to the transmembrane zinc transporter ZrfC and participate in zinc homeostasis [76]. The metallothionein CrdA was involved in copper homeostasis and responded to high zinc stimulation in *A. fumigatus* (Figure 1) [52]. Deletion of the *crdA* gene did not affect zinc tolerance, but the overexpression of CrdA partially rescued the zinc sensitivity of *zrcA* or *crpA* deletion mutants, which suggests that CrdA is also involved in zinc homeostasis. However, whether CrdA is also a metallothionein of zinc is not clear, and more studies are needed to verify this hypothesis.

The metal chaperone protein Mtm1 is a manganese-trafficking factor that is essential for the maturation of mitochondrial MnSOD in *S. cerevisiae* [77] and Mtm1 is important for zinc homeostasis [78]. The deletion of *mtm1* reduced the transcription of *zrt1* and *zap1*, which decreased intracellular zinc accumulation but significantly increased zinc accumulation in vesicles due to the upregulation of *zrc1*. Notably, MtmA, the homolog of Mtm1, is critical for hyphal growth in *A. fumigatus*, and further studies suggest that this may be due to the detoxification effect of zinc [79]. The chelation of zinc obviously rescued growth defects caused by the suppression of MtmA, but its function as a zinc chaperone has not been confirmed. Therefore, its role in zinc trafficking needs to be further investigated in *A. fumigatus*. The COG0523 family’s proteins likely play a role in zinc metalloprotein maturation and participate in zinc homeostasis [80], but three homologs of MchA/B/C in *A. fumigatus* have not been investigated.

## 3. Zinc Homeostasis for the *A. fumigatus* Virulence

Since host lung tissue provides a slightly alkaline zinc-limited environment to inhibit microbial growth, fungal pathogens have a highly coordinated mechanism to regulate zinc homeostasis to prevent zinc deficiency and thus promote their survival. As mentioned previously, *A. fumigatus* uses different types of proteins to regulate zinc homeostasis, including zinc-responsive transcription factors (ZafA, PacC and AceA) and zinc transporters (import/export), which are important in the virulence of *A. fumigatus* [52,71,73]. Although double deletion mutations in zinc importers ZrfA and ZrfB exhibited normal virulence, ZrfC was critical to the virulence of *A. fumigatus* since the *ΔzrfC* mutant showed significantly reduced virulence in an immunocompromised murine model of invasive aspergillosis (IA) [30]. Triple deletion mutation of zinc importers ZrfA/B/C produced non-virulence in *A. fumigatus.* The role of ZrfC protein in fungal virulence primarily depends on its special long N-terminus on the extracellular side of the membrane to obtain Zn directly from the lungs, which is not present in the acidic transporters ZrfA or ZrfB [30]. Because *zrfA*, *zrfB* and *zrfC* are highly induced in a ZafA-dependent manner in a zinc-limited environment, ZafA is also considered a virulence factor for *A. fumigatus* [71]. The deletion mutant of ZafA is avirulent compared to the parental wild type in the IA immunocompromised mouse model [71]. Transcription factor Zap1 promoted the expression of importers Zrt1 and Zrt2 in the plasma membrane in *S. cerevisiae* and induced the vacuole transporter Zrt3 to mobilize stored zinc in response to zinc deficiency [69,70]. ZrtF (homolog of Zrt3) is a conserved protein in *Aspergillus* species, but its role in virulence as a zinc transporter on vacuoles in *A. fumigatus* has not been investigated. Similar to ZafA, another transcription factor PacC was necessary for the virulence of *A. fumigatus* because PacC deletion significantly reduced virulence [73]. In addition, transcription factor AceA is involved in copper and zinc detoxification by regulating transporters *crpA* and *zrcA*, and it is required for *A. fumigatus* virulence [52]. AceA is involved in the virulence of *A. fumigatus* primarily by regulating the detoxification of copper by *crpA*, but it is not known whether the involvement of AceA in the detoxification of Zn contributes to virulence. However, it has been shown that host macrophages provide a mechanism of zinc poisoning against *Mycobacterium tuberculosis* [81]. This mechanism has not been described as a defense mechanism against fungal pathogens, and future intensive studies on this mechanism in fungi are necessary.

## 4. Potential Antifungal Targets of Zinc Homeostasis Regulators and Future Perspectives

With the recent increase in drug-resistant strains, antifungal drugs used to treat fungal pathogens are not sufficiently effective for inhibiting the growth of *A. fumigatus* as a monotherapy or in combination, and clinical mortality remains high [6]. Therefore, the discovery and development of a new generation of antifungal drugs targeting *A. fumigatus-*specific metabolic pathways are necessary as an alternative to the proteins targeted by classic antifungals. Since *A. fumigatus* has evolved an effective zinc-acquisition mechanism to counteract zinc limitation, we propose a therapeutic strategy based on preventing zinc acquisition to inhibit *A. fumigatus* growth. The zinc uptake by *A. fumigatus* in the slightly alkaline environment of the host is primarily mediated by the zinc importer ZrfC [30]. Therefore, ZrfC is a potential drug target, and any compound that impairs the function of ZrfC would predictably inhibit the growth of *A. fumigatus* in the host. ZrfC primarily relies on a specific N-terminal uptake of extracellular zinc, and there is no homolog in humans [30]. Thus, any compound targeting the N-terminal end of ZrfC to inhibit its function would be theoretically effective. The zinc-responsive transcription factor ZafA in *A. fumigatus* is essential for the regulation of zinc homeostasis and virulence, and *A. fumigatus* uses ZafA to activate zinc importers ZrfA, ZrfB and ZrfC, which prevents zinc deficiency [13,72]. Notably, the *ΔzafA* mutant exhibited lower virulence than the *ΔzrfC* mutant in an immunocompromised mouse model, which suggests that ZafA plays a major role in the virulence of *A. fumigatus* [71]. Similar to ZrfC, the ZafA protein is only distributed in fungi and has no homolog in humans. In addition, due to the presence of C_2_H_2_-type zinc fingers of transcription factors in the host, the inhibition of ZafA should be based on a specific blockade of its transcriptional activation domain rather than interference with its C_2_H_2_-type zinc finger-dependent DNA binding domain.

Recent academic research and biomedical companies have effectively used fragment-based drug discovery to investigate selective inhibitors against highly conserved effective targets [82]. Although small fragments screened in this manner have the potential to bind to non-conserved sites of the target, selective inhibitors may be obtained by iterative cycles of chemical optimization guided by structural information via in vitro enzymatic or in vivo inhibition assays [83,84]. Because the expression of zinc acquisition genes is essential for the survival and virulence of fungal pathogens, disruption of the function of zinc acquisition genes would predict the following beneficial effects. On the one hand, disruption of zinc acquisition genes would lead to a severe deficiency in zinc resulting in fungistatic and/or fungicidal effects on fungal pathogens. On the other hand, the combination with current antifungal drugs could improve efficacy, promote fungal killing and prevent the emergence of drug resistance.

Taken together, the regulation of zinc homeostasis required for *A. fumigatus* infection may provide a promising therapeutic target. In future studies, new compounds specifically blocking the regulation of zinc homeostasis in *A. fumigatus* could be tested for potential as antifungals. Moreover, the molecular characterization of fungal-specific zinc-responsive transcription factor ZafA and zinc importer ZrfC must be further explored and used as potential drug targets for screening novel antifungal drugs through fragment-based drug discovery or other drug-screening strategies.

## Figures and Tables

**Table 1:** Zinc homeostasis-related genes of *Aspergillus fumigatus* ZIP protein family.

Systematic Name	Gene Name *	Description	Location	References
AFUB_079250	*zrfA(zrt1)*	Plasma membrane zinc transporter	Plasma membrane	[46]
AFUB_020930	*zrfB(zrt2)*	Plasma membrane zinc transporter	Plasma membrane	[46]
AFUB_066680	*zrfC*	Plasma membrane zinc transporter	Plasma membrane	[30,47]
AFUB_097050	*zrfD*	Putative zinc importer	Plasma membrane (Putative)	[41]
AFUB_083560	*zrfE*	Putative zinc importer	Plasma membrane (Putative)	[41]
AFUB_024650	*zrfF(zrt3)*	Putative zinc importer	Vacuole (Putative)	Predicted in this study
AFUB_018540	*zrfG(yke4)*	Putative zinc importer	Endoplasmic reticulum (Putative)	Predicted in this study
AFUB_027750	*zrfH(atx2)*	Putative zinc importer	Golgi (Putative)	Predicted in this study

Gene name *, within parentheses orthologs in *S. cerevisiae* are indicated.
